# Repetitive T1 Imaging Influences Gray Matter Volume Estimations in Structural Brain Imaging

**DOI:** 10.3389/fneur.2021.755749

**Published:** 2021-10-28

**Authors:** Gregor Broessner, Isabel Ellerbrock, Mareike M. Menz, Florian Frank, Michael Verius, Christian Gaser, Arne May

**Affiliations:** ^1^Department of Systems Neuroscience, University Medical Center Hamburg-Eppendorf, Hamburg, Germany; ^2^Department of Neurology, Medical University of Innsbruck, Innsbruck, Austria; ^3^Department of Neuroradiology, Medical University Innsbruck, Innsbruck, Austria; ^4^Departments of Neurology and Psychiatry, Jena University Hospital, Jena, Germany

**Keywords:** voxel-based morphometry (VBM), structural plasticity, pain, Nociception, morphometry

## Abstract

Voxel-based morphometry (VBM) is a widely used tool for studying structural patterns of brain plasticity, brain development and disease. The source of the T_1_-signal changes is not understood. Most of these changes are discussed to represent loss or possibly gain of brain gray matter and recent publications speculate also about non-structural changes affecting T_1_-signal. We investigated the potential of pain stimulation to ultra-short-term alter gray matter signal changes in pain relevant brain regions in healthy volunteers using a longitudinal design. Immediately following regional nociceptive input, we detected significant gray matter volume (GMV) changes in central pain processing areas, i.e. anterior cingulate and insula cortex. However, similar results were observed in a control group using the identical time intervals but without nociceptive painful input. These GMV changes could be reproduced in almost 100 scanning sessions enrolling 72 healthy individuals comprising repetitive magnetization-prepared rapid gradient-echo (MPRAGE) sequences. These data suggest that short-term longitudinal repetitive MPRAGE may produce significant GMV changes without any intervention. Future studies investigating brain plasticity should focus and specifically report a consistent timing at which time-point during the experiment the T_1_-weighted scan is conducted. There is a necessity of a control group for longitudinal imaging studies.

## Introduction

Voxel-based morphometry (VBM) is a widely used non-invasive imaging tool (1–3) to investigate patterns of structural brain change either between cohorts, e.g. various disease entities vs. healthy controls (4, 5) or longitudinal, e.g. before and after a specific learning task such as juggling or learning (6, 7). VBM essentially involves voxel-wise statistical analysis of pre-processed structural T_1_-weighted MR images. Longitudinal VBM analyses provide the possibility to compare patterns of brain change over a distinct period.

VBM has been increasingly used to describe differences in brain structure between chronic pain patients and controls (8). A striking feature of all of these studies is the fact that gray matter changes (9) were not randomly distributed, but concerned defined and functionally highly specific brain areas – namely, areas involvement of supraspinal nociceptive processing (10). Most of these changes are discussed to represent damage, loss or even gain of brain gray matter, reinforcing the idea of measuring brain plasticity by T_1_-weighted signal differences using VBM. An abundance of human studies, including several of our own, report morphologic alterations of the brain in areas responsible for the transmission of pain in patients suffering from chronic pain conditions such as phantom pain, chronic back pain, neuropathic pain, irritable bowel syndrome, fibromyalgia and two types of frequent headaches (6, 11–13). Several recent longitudinal studies involved learning paradigms and nearly all of them attribute their findings to be (a) specific for the respective learning paradigm and (b) to imply some kind of change on cell-level, mostly synaptogenesis (14, 15). However, at least three recent studies question the common belief that brain changes observed through VBM are always based on changes in cellular or neuronal structure of gray matter respectively. Tost et al. demonstrated GMV changes after single dose administration of a neuroleptic drug, suggesting interpretation of these morphological short-term changes as synaptogenic alterations (16). More recently, Franklin et al. identified short-term effects of a single dose of medication (baclofen) and demonstrated a partial overlap of these changes with concurrent changes in perfusion f-MRI (3). The authors concluded that T_1_-relaxation time for arterial blood and gray matter might not be clearly distinguishable and therefore structural findings could be at least partially attributed to changes in blood flow suggesting that the observed changes have to be interpreted as T_1_-weighted signal differences and not as gray matter alterations (3). Tardif et at. demonstrated that increased blood volume from vasodilation during hypercapnia, induced by hyperventilation, is associated with an overestimation of cortical thickness (1.85%) and gray matter volume (3.32%), and that both changes in O2 concentration and blood volume [i.e. cerebral blood flow (CBF)] lead to changes in the T_1_ value of tissue (17).

As the central mechanisms of pain processing are well understood in experimental as well as chronic pain, we chose pain stimulation in healthy participants as a model to study *in vivo* non-pharmacological short-term morphometric changes in humans. In this study we primarily hypothesized that electrical high intensity pain stimulation could lead to ultra-short term pain dependent changes in brain structure using high-field magnetic resonance imaging (MRI). Only upon a closer look at the results obtained from a control group without any electrical painful stimulation we changed the study design and further developed our hypothesis toward scanner environment or loco-regional blood flow fluctuations being responsible for the detected GMV changes.

## Materials and Methods

**Figure 1:**
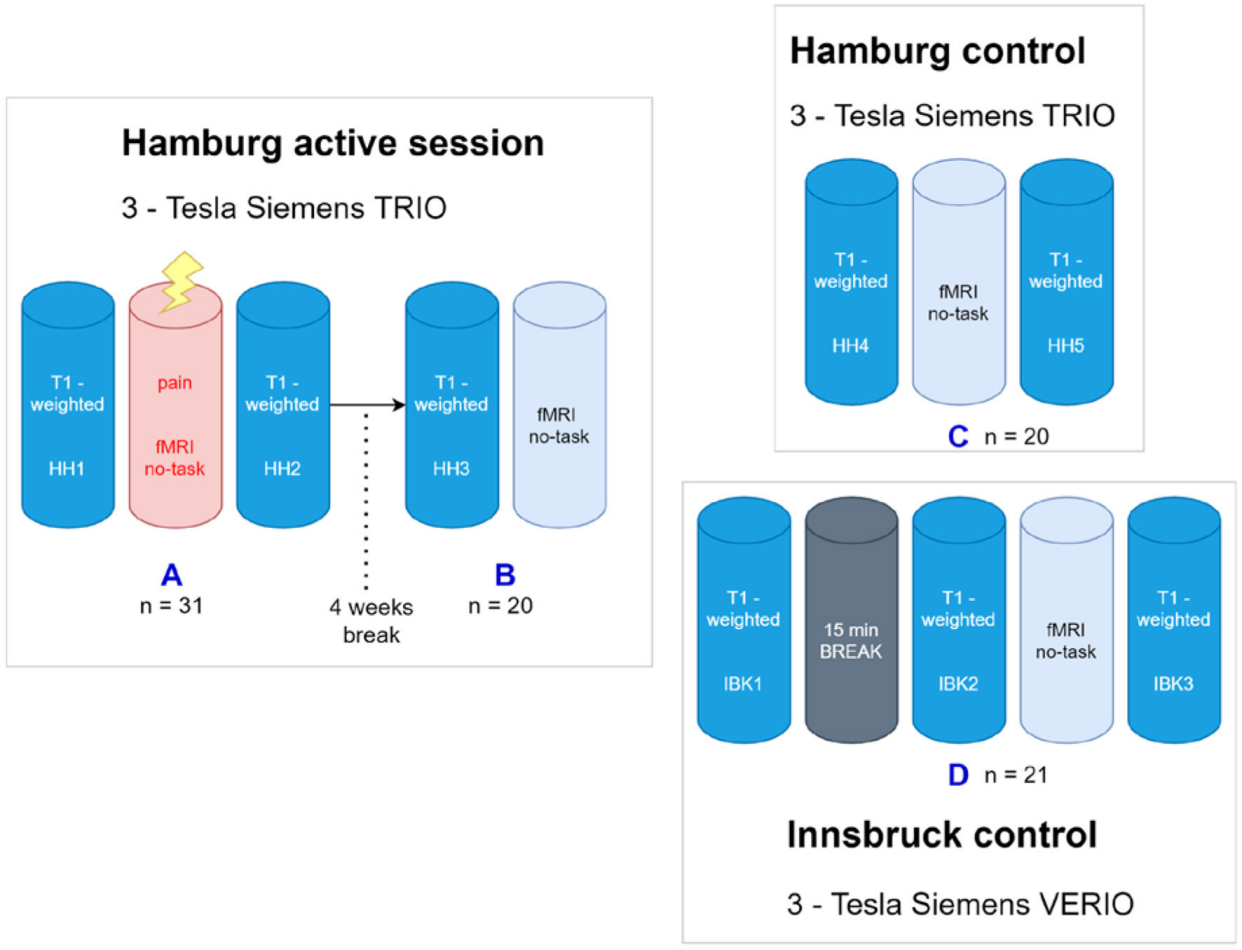
Overview about the project. **(A)** The first scanning paradigm was carried out in Hamburg using a 3-Tesla Siemens Trio MRI. After T1-weighted image acquisition an fMRI including a nociceptive task was done, again followed by T1-weighted image acquisition. **(B)** After four wk, the *post hoc* image acquisition comprised another T1-weighted followed by a (taskless) fMRI session of identical length as the first (painful) fmri session four wk earlier. The control paradigm was carried out using two different, but comparable MRI scans in Hamburg and Innsbruck. **(C)** The Hamburg control comprised a fMRI acquisition engulfed by two structural T1WI scans. **(D)** For the Innsbruck control paradigm, three T1-weighted image acquisitions were performed, the first one followed by a rest period without scanning but the participants stayed in the scanner, and, in the same session, the second one with an (again taskless) fMRI session of identical length. SHH1, Sequence 1 at Hamburg Scanner; SIBK1, Sequence 1 at Innsbruck Scanner.

A detailed overview of the scanning sessions including the 4 projects (A-D) can be found in Figure 1. Projects A, B, and C were scanned in Hamburg, Germany on a Siemens® 3T TRIO Scanner. Data of project D was obtained at Innsbruck (Siemens® 3T VERIO). Participants of project A & B are identical. Participants of project C & D represent new and independent groups.

All subjects were right-handed and male. They were recruited locally and all systematically screened by an experienced full-time neurologist and pain specialist to ensure they were free of any lifetime history of neurological or psychiatric illness. Subjects with acute or chronic pain related disorders were excluded. Consumption of any medication (including pain medication) seven days prior to scanning led to exclusion. All participants had normal or corrected-to-normal vision and gave written informed consent following a detailed explanation of study procedures. The study was approved by the local ethics committee (Ärztekammer Hamburg PV3562) and all participants gave written informed consent.

### Project A (Hamburg, *n* = 31)

Prior to the MRI experiment, the 31 healthy subjects (mean age 26.87, SD ± 4.69) were trained to answer the visual analog scale (VAS) by moving a slider via button press. The rating scales were presented via back-projection through a 45° mirror placed atop the head coil. For pain stimulation an electrode was placed at the dorsum of the left hand between the extensor tendon of the index and middle finger. A first eight min T_1_-weighted acquisition (S_HH1_) was performed followed by an immediate calibration of the individual pain threshold. This calibration was achieved by increasing an electric current stepwise until volunteers reported painful but bearable stimulation (pain rating of 8 on a 1–10 point scale). After calibrating the pain threshold subjects underwent the pain stimulation paradigm simultaneously with fMRI acquisition. The paradigm (approx. 20 min) consisted of 15 blocks, each of them comprising a repetitive short-term electrical pain stimulus (Digitimer constant current stimulator DS7A®, Digitimer Ltd, Hertfordshire, AL7 3BE, England) and a subsequent interval without stimulation in which the subjects could rate pain intensity and unpleasantness. During the stimulation paradigm participants were presented a gray fixation cross via the 45° mirror. Finally, a second T_1_-weighted MPRAGE (S_HH2_) was performed with identical parameters as the first. Total scan duration was approx. 40 min.

Task presentation and recording of behavioral responses was performed with Presentation 14.2 (NeuroBehavioral Systems, Inc.).

The experimental sequence can be summarized as: First T_1_-weighted MPRAGE (S_HH1_) followed by fMRI with painful stimuli and second T_1_-weighted MPRAGE (S_HH2_).

### Project B (*n* = 20)

To test for a putative bias introduced by pain expectation of the volunteers preceding the task (during S_HH1_) a *post-hoc* analysis after a time frame of four wk was conducted. Therefore 20 randomly chosen volunteers of the original study population were invited to participate again in a second scanning session. This short session only consisted of an MPRAGE (S_HH3_) and a 7.5 min fMRI baseline measurement with identical scanner settings as described above. Prior to scanning all volunteers were again screened for meanwhile neurologic or psychiatric disorders including pain symptoms and medication by the same study investigator. To ensure that volunteers did neither expect nor fear possible painful stimulation, there was no electrode attached to the subjects' hand.

### Project C (*n* = 20)

Prior to the MRI experiment, 20 new and independant healthy subjects (mean 26.3, SD ± 4.39) were trained to answer a visual analog scale by moving a slider via button press to test for awareness and unpleasantness similarly to project A, but without pain rating. Volunteers were previously informed that they would serve as a control group to a pain study. To ensure that volunteers did not expect nor fear possible painful stimulation, there was no electrode attached to the subjects' hand.

This session consisted of an MPRAGE (S_HH4_) followed by an fMRI measurement including awareness testing again followed by an MPRAGE (S_HH5_) with identical scanner settings as described above.

### Project D (Innsbruck, *n* = 21)

In total 25 healthy subjects were scanned. 21 subjects (mean age 24, SD ± 1.47) were included in the final VBM analysis (3 subjects were excluded due to motion artifacts, 1 subject terminated the scanning session prematurely).

Prior to the MRI experiment, subjects were trained to answer a visual analog scale by moving a slider via button press to test for awareness and in analogy to project A which included the pain rating.

This session consisted of a first seven min MPRAGE (S_IBK1_) followed by a resting period without scans (REST, 15 min) then a second seven min MPRAGE (S_IBK2_) and an 18 min fMRI measurement including awareness testing again followed by an identical MPRAGE (S_IBK3_). In the resting phase the subjects had to remain in the scanner and were asked not to move nor to fall asleep. The 15-block fMRI paradigm in this cohort was free of any pain stimulation but subjects were alternatingly presented with a fixation cross for two min followed by rating scales to assess unpleasantness and awareness to monitor vigilance. To ensure that volunteers did neither expect nor fear possible painful stimulation, there was no electrode attached to the subjects' hand.

### Acquisition of Magnetic Resonance Images in Hamburg (Projects A-C)

Magnetic resonance imaging (MRI) was performed on a 3-Tesla whole body system (Siemens®, Erlangen Germany), using a standard 32-channel receive-only head coil. Earplugs and foam padding were used to minimize background noise and head motion. For structural MRI, a T1-weighted, three-dimensional magnetization-prepared rapid gradient-echo (MPRAGE) sequence was used with the following parameters: TI = 1100 ms, TR = 2300 ms, TE = 3.0 ms, flip angle = 9°, 192 × 256 × 240 mm^3^ field-of-view, and voxel resolution = 1.0 mm × 1.0 mm × 1.0 mm^3^. These settings were kept identical for all structural scanning sequences.

For functional MRI, T2^*^-weighted images were collected parallel to the anterior commissure – posterior commissure (AC–PC) plane using an echo-planar imaging (EPI) sequence with the following parameters for all EPI acquisitions: TR = 2620 ms, TE = 30 ms, flip angle = 80°, 40 slices with a gap of 1.0 mm in-between, 222 × 222 mm^2^ field-of-view, and voxel resolution = 3.0 × 3.0 × 3.0 mm^3^.

### Acquisition of Magnetic Resonance Images in Innsbruck (Project D)

For data acquisition in Innsbruck, protocol parameters were matched and kept as close as possible. Magnetic resonance imaging (MRI) was performed on a 3-Tesla whole body scanner (Siemens, Erlangen Germany), using a standard 12-channel receive-only head coil. Earplugs and foam padding were used to minimize background noise and head motion. For structural MRI, a T_1_-weighted, three-dimensional magnetization-prepared rapid gradient-echo (MPRAGE) sequence was used with the following parameters: TI = 1100 ms, TR = 2300 ms, TE = 2.98 ms, flip angle = 9°, 192 x 256 x 240 mm^3^ field-of-view, and voxel resolution = 1.0 mm × 1.0 mm × 1.0 mm^3^.

For functional MRI, T2^*^-weighted images were collected parallel to the anterior commissure – posterior commissure (AC–PC) plane using an echo-planar imaging (EPI) sequence with the following parameters: TR = 2630 ms, TE = 30 ms, flip angle = 80°, 40 slices with a gap of 1.0 mm in-between, 222 × 222 mm^2^ field-of-view, and voxel resolution = 3.0 × 3.0 × 3.0 mm^3^. Prior to data acquisition a manual prescan was performed followed by shimming.

The parameters and settings were identical for all subjects and measurements throughout the study.

### Data Processing and Image Analysis

Data preprocessing and analysis was performed with SPM12 (Welcome Department of Cognitive Neurology, London, UK) running under Matlab (R2010a, Mathworks) and the Computational Anatomy Toolbox (CAT12 r1740) toolbox (http://www.neuro.uni-jena.de/cat). Prior pre-processing all scans were manually reoriented to the anterior commissure. For preprocessing we used the CAT12 longitudinal pipeline for detecting short-term changes, which consists of the following steps:

1. Longitudinal rigid registration: The images of all time points of one subject are registered to the midpoint average image, which is created by the average of time and space across all time points. We applied an inverse-consistent registration that guarantees that all time points are corrected by the same amount of rotations and translations. This will prevent interpolation effects that can occur if all images are registered only to the first time point. Thus, the order of the time points will not affect the preprocessing and analysis. Furthermore, a bias field correction between the images was also applied.
2. Segmentation of the mid-point average image: The mid-point average image was corrected for bias field inhomogeneities and segmented into gray matter (GM), white matter (WM) and cerebrospinal fluid (CSF) by applying an adaptive maximum a posteriori estimation approach (18) and a partial volume effects model (19).
3. Segmentation of the images of all time points: The segmentation approach is the same as in step 2. However, while the mid-point average image is only segmented in order to estimate the non-linear spatial transformation to the template image the images of all time points in step 3 are segmented for further analysis.
4. Estimation of non-linear spatial transformation: The segmentation of the mid-point average image is used to estimate the DARTEL non-linear spatial transformations that are necessary to deform the segmented image to the DARTEL template that is provided with CAT12 and defined in MNI space (1).
5. Write spatially registered images: The deformations that were estimated in step 4 using the mid-point average image are finally applied to the segmented images of all time points.

Finally, all spatially registered GM images were smoothed with 8 mm full-width-at-half-maximum (FWHM) isotropic Gaussian kernel. All data passed the quality check that is implemented in CAT12 and obtains measures of noise, bias and overall image quality.

We have used a repeated measures ANOVA with the factors time, group and scanning site for statistical analysis that takes the longitudinal study design into account. GM images of all experiments (A, C, and D) were combined in one model where the differences between the time points were compared for each experiment separately. Total intracranial volume (TIV) for each time point was used as a nuisance variable to remove any effects due to different brain size. Although no TIV changes are expected between time points, we used it as a nuisance variable to detect relative changes between time points rather than absolute changes that would be observed without TIV correction.

Finally, we combined the comparison between the time points for all experiments using a global conjunction analysis. This analysis tests where the differences between the time points were consistently high and jointly significant across all experiments. Our intention for applying this test was to find common differences between the time points across all experiments.

For all analyses, the significance threshold was set to *P* < 0.05 and family-wise error (FWE) corrected for multiple comparisons at the voxel level using Gaussian Random Field theory.

Only clusters with a minimum cluster size of 12 voxels (according to the expected numbers of voxels per cluster) are reported. Furthermore, we applied an absolute threshold of 0.1 for the analysis to guarantee that only gray matter areas are analyzed.

In order to check that there is no dependency of our longitudinal preprocessing from the order of the time points we again used the data of Experiment D. We selected the three time points for preprocessing in the order 1-2-3, 2-1-3, and 3-1-2 and preprocessed and analyzed the data independently using these different order selections.

In order to test any dependencies of the used segmentation and spatial registration approaches on our results, we additionally applied another two VBM pipelines to our data.

We used the registered images of step 1 (longitudinal rigid registration) and segmented and spatially registered the data with the segmentation and Dartel registration of SPM12 and the VBM pipeline in FSL 6 (FSL-VBM v1.1) using the default settings, except for the skull-stripping with FSL6-BET that was applied with parameters optimized for the used data.

We have also tried the Longitudinal Registration Toolbox in SPM12 that determines the changes between the time points using the Jacobian determinant of the deformations. However, here the registration regularization is changed depending on the time between scans. Our time intervals of a few minutes between scans lead to an extremely high regularization, which ultimately prevents the detection of such short-term changes. Thus, this approach was not included in the analysis.

## Results

In total 72 healthy volunteers (in 96 scanning sessions) were consecutively enrolled in the study that comprised 4 scanning sessions (Projects A-D, Figure 1). Because we observed more statistical power and larger effects in the sample of project A (*n* = 31), we always used randomly selected 20 volunteers in each sample for the structural data to obtain a balanced design in terms of sample size and statistical power. We therefore only report the results for this balanced design that allows a fair and reliable comparison between all experiments without bias due to different sample sizes.

In none of these projects we found a significant difference in the values of total intracranial volume or total gray matter volume between sessions.

### Hamburg (3T Siemens® TRIO), Projects A to C

#### Project A (fMRI+PAIN, Hamburg)

Reduced local gray matter volume after pain stimulation (S_HH2_ < S_HH1_) was observed in several areas including bilateral caudate, left frontal and cingulate gyrus.

Volunteers showed a significantly larger local gray matter volume after pain stimulation (S_HH1_ < S_HH2_) in several occipital and parietal areas, including the left parietal operculum, the left middle and posterior cingulate gyrus, and the precuneus in both hemispheres.

**Figure 2:**
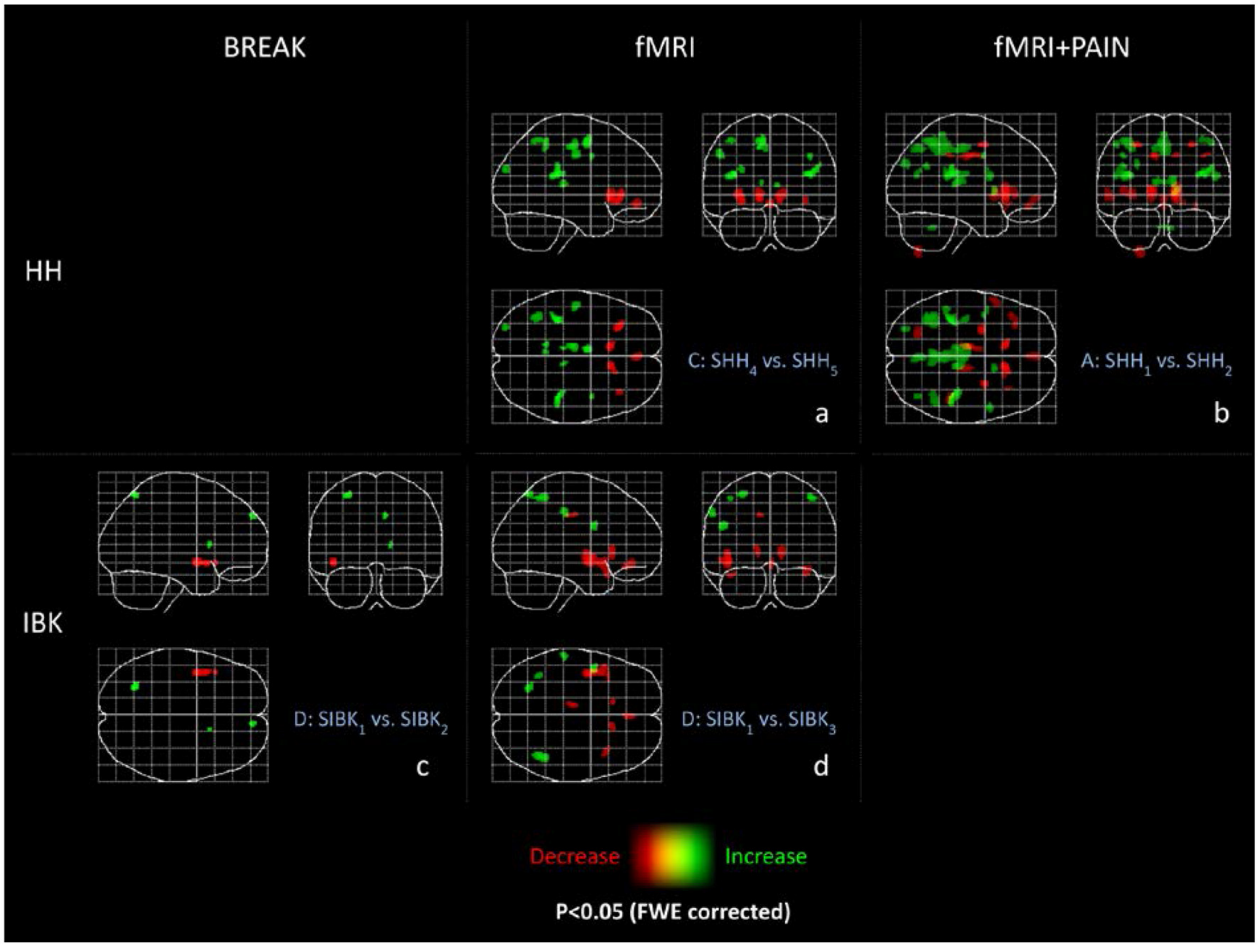
Maximum intensity projections (MIP) of all experiments. These maximum intensity projections summarize the T-statistics of all experiments. Indicated are increases (green) and decreases (red) in GM volume. All results are thresholded at *p* < 0.05 FWE-corrected and only clusters are displayed with a minimum cluster size of 36 voxels (according to a cluster size threshold of *p* < 0.05) as MIP. The abbreviations of the experiments are according to the naming scheme in Figure 1.

Please refer to Figure 2b and Table 1 for full results.

**Table 1:** MNI-coordinates, maximum *T*-value, cluster size (in voxel) above threshold and anatomical site (*p* < 0.05 FWE-corrected) are reported for GM changes in experiment A of the Hamburg data (fMRI+PAIN).

***T*-Value**	**Size**	**xyz [mm]**	**Overlap**	**Atlas region**
**GM Decrease (A: fMRI+PAIN HH1 vs. HH2)**				
14, 26	272	12, 21, 0	63%	Right Caudate
			23%	Right Cerebral White Matter
			11%	Right Lateral Ventricle
13, 66	139	−12, 22, 4	63%	Left Caudate
			22%	Left Lateral Ventricle
			14%	Left Cerebral White Matter
12, 17	76	−10, −16, 39	100%	Left Middle Cingulate Gyrus
12	36	−26, −3, 50	92%	Left Middle Frontal Gyrus
11, 59	36	28, 0, 50	100%	Right Middle Frontal Gyrus
8, 51	34	12, −20, 39	100%	Right Middle Cingulate Gyrus
8, 25	129	−33, 33, 2	47%	Left Frontal Operculum
			36%	Left Orbital Part of the Inferior Frontal Gyrus
			17%	Left Triangular Part of the Inferior Frontal Gyrus
8, 07	132	−24, −68, −58	100%	Left Cerebellum Exterior
7, 98	22	44, −38, 40	100%	Right Supramarginal Gyrus
7, 89	89	−57, 10, −2	57%	Left Opercular Part of the Inferior Frontal Gyrus
			20%	Left Temporal Pole
			12%	Left Frontal Operculum
6, 92	104	0, 51, −4	30%	Right Superior Frontal Gyrus Medial Segment
			25%	Left Medial Frontal Cortex
			17%	Right Medial Frontal Cortex
			16%	Left Superior Frontal Gyrus Medial Segment
			12%	Left Anterior Cingulate Gyrus
6, 2	8	32, 24, −10	88%	Right Posterior Orbital Gyrus
			12%	Right Lateral Orbital Gyrus
**GM Increase (A: fMRI+PAIN HH1 vs. HH2)**				
−13, 25	128	−34, −33, 21	86%	Left Parietal Operculum
−13, 19	116	39, −32, 22	88%	Right Parietal Operculum
−9, 68	953	−2, −51, 51	32%	Left Precuneus
			25%	Right Precuneus
−9, 4	165	9, −28, 12	25%	Right Thalamus Proper
−9, 36	288	−39, −54, 56	49%	Left Superior Parietal Lobule
			48%	Left Angular Gyrus
−9, 04	51	51, 4, 18	100%	Right Precentral Gyrus
−8, 83	19	−40, −58, 28	100%	Left Angular Gyrus
−8, 53	125	2, −68, 46	65%	Right Precuneus
			35%	Left Precuneus
−8, 36	49	12, 9, 4	55%	Right Caudate
−8, 29	14	−40, −2, 39	100%	Left Precentral Gyrus
−7, 72	34	−48 −39 51	100%	Left Supramarginal Gyrus
−7, 56	68	6, −52, −33	28%	Right Cerebellum Exterior
			26%	Right Cerebellar Vermal Lobules VIII–X
−7, 56	34	−38, −20, 22	94%	Left Central Operculum
−7, 51	27	40, −68, 21	100%	Right Middle Occipital Gyrus
−7, 49	114	46, −50, 54	66%	Right Angular Gyrus
−7, 46	59	−27, −80, 32	73%	Left Middle Occipital Gyrus
			27%	Left Superior Occipital Gyrus
−7, 09	22	−48, 3, 20	100%	Left Precentral Gyrus

*All clusters in gray matter with a minimum cluster size of 12 voxels are reported according to an additional cluster size threshold of p < 0.05. The percentage overlap (a minimum of 25% is shown) of the atlas regions is derived from the Neuromorphometrics Atlas*.

#### Project B

The comparison of S_HH1_ with the *post-hoc* structural images (S_HH3_), i.e. comparing two T_1_ images without preceding T_1_ or functional scans, revealed neither increase nor decrease in local gray matter volume above threshold (data not shown).

#### Project C (fMRI, Hamburg)

Reduced local gray matter volume without pain stimulation (S_HH5_ < S_HH4_) was observed among others bilaterally in the caudate, the inferior frontal gyrus, the medial frontal cortex, and in the left anterior insula.

Significantly larger local gray matter volume (S_HH4_ < S_HH5_) could be detected in volunteers without pain stimulation bilaterally in the parietal operculum, thalamus, the left angular and occipital gyrus, superior parietal lobule, and the motor cortex.

Please refer to Figure 2a and Table 2 for detailed results.

**Table 2:** MNI-coordinates, maximum *T*-value, cluster (in voxel) size above threshold and anatomical site (*p* < 0.05 FWE-corrected) are reported for GM changes in the experiments C of the Hamburg data (fMRI).

***T*** **-Value**	**Size**	**xyz [mm]**	**Overlap**	**Atlas region**
**GM Decrease (C: fMRI HH4 vs. HH5)**				
10, 62	134	−10, 20, −3	55%	Left Caudate
			27%	Left Cerebral White Matter
10, 16	131	−28, 30, −2	69%	Left Orbital Part of the Inferior Frontal Gyrus
9, 17	128	10, 21, −3	53%	Right Caudate
7, 34	33	36, 28, −6	91%	Right Orbital Part of the Inferior Frontal Gyrus
7, 17	71	0, 46, −9	35%	Left Medial Frontal Cortex
**GM Increase (C: fMRI HH4 vs. HH5)**				
−10, 77	66	−36, −34, 21	86%	Left Parietal Operculum
−10, 56	76	40, −33, 21	82%	Right Parietal Operculum
−9, 91	91	−8, −3, 54	100%	Left Supplementary Motor Cortex
−9, 27	62	−12, −16, 48	52%	Left Supplementary Motor Cortex
			48%	Left Precentral Gyrus Medial Segment
−8, 06	21	−12, −45, 54	100%	Left Precuneus
−7, 76	14	40, 0, 39	100%	Right Precentral Gyrus
−7, 54	77	−40, −54, 56	69%	Left Angular Gyrus
			30%	Left Superior Parietal Lobule
−7, 49	81	−51, −15, 42	100%	Left Postcentral Gyrus
−7, 3	44	−28, −87, 26	86%	Left Middle Occipital Gyrus
−6, 88	12	−9, −28, 12	33%	Left Thalamus Proper
−6, 86	13	8, −28, 9	85%	Right Thalamus Proper
−6, 61	27	−4, −46, 46	100%	Left Precuneus

*All clusters in gray matter with a minimum cluster size of 12 voxels are reported according to an additional cluster size threshold of p < 0.05. The percentage overlap (a minimum of 25% is shown) of the atlas regions is derived from the Neuromorphometrics Atlas*.

### Project D (fMRI, Innsbruck)

Reduced local gray matter volume without pain stimulation (S_IBK3_ < S_IBK1_) was observed bilaterally in the caudate, insula, and temporal pole, the left posterior orbital and cingulate gyrus. Please refer to Figure 2d and Table 3 for detailed results.

**Table 3:** MNI-coordinates, maximum *T*-value, cluster size (in voxel) above threshold and anatomical site (*p* < 0.05 FWE-corrected) are reported for GM changes in experiment D of the Innsbruck data (BREAK).

***T*** **-Value**	**Size**	**xyz [mm]**	**Overlap**	**Atlas region**
**GM Decrease (D: fMRI IBK1 vs. IBK3)**				
10, 36	382	−42, −2, −8	45%	Left Anterior Insula
7, 74	21	−10, −18, 39	100%	Left Middle Cingulate Gyrus
7, 31	27	−14, 22, 4	56%	Left Caudate
7, 1	69	12, 21, 2	83%	Right Caudate
6, 92	71	36, 16, −20	82%	Right Anterior Insula
6, 81	66	2, 39, −10	65%	Right Medial Frontal Cortex
6, 35	18	−40, 18, −22	78%	Left Temporal Pole
**GM Increase (D: fMRI IBK1 vs. IBK3)**				
−8, 79	97	42, −51, 57	47%	Right Angular Gyrus
			35%	Right Superior Parietal Lobule
−8, 19	52	−46, 3, 28	100%	Left Precentral Gyrus
−8, 02	46	−27, −63, 60	100%	Left Superior Parietal Lobule
−7, 51	53	−58, −27, 40	100%	Left Supramarginal Gyrus
−6, 6	25	−40, −51, 56	56%	Left Angular Gyrus
			44%	Left Superior Parietal Lobule
**GM Increase (D: fMRI IBK1 vs. IBK2)**				
6.17	128	−42, 0, −9	52%	Left Anterior Insula
5.23	9	−44, 20, −8	100%	Left Orbital Part of the Inferior Frontal Gyrus
**GM Decrease (D: fMRI IBK1 vs. IBK2)**				
5.96	40	−30, −63, 58	100%	Left Superior Parietal Lobule
5.61	22	9, 57, 38	100%	Right Superior Frontal Gyrus
5.17	9	15, 14, 9	100%	Right Caudate

*All clusters in gray matter with a minimum cluster size of 12 voxels are reported according to an additional cluster size threshold of p < 0.05. The percentage overlap (a minimum of 25% is shown) of the atlas regions is derived from the Neuromorphometrics Atlas*.

Significantly larger local gray matter volume (S_IBK1_ < S_IBK3_) could be detected in volunteers without pain stimulation bilaterally in the angular gyrus, the superior parietal lobe, the supramarginal gyrus, and the left precentral gyrus.

### Project D (Test for Non-dependency From Order of Selected Time Points)

We obtained almost exactly the same results as in section Project D (fMRI, Innsbruck, Figure 2d) for the three different orders of selecting the time points (results not shown).

### Global Conjunction Across All Three Experiments (A+C+D)

**Figure 3:**
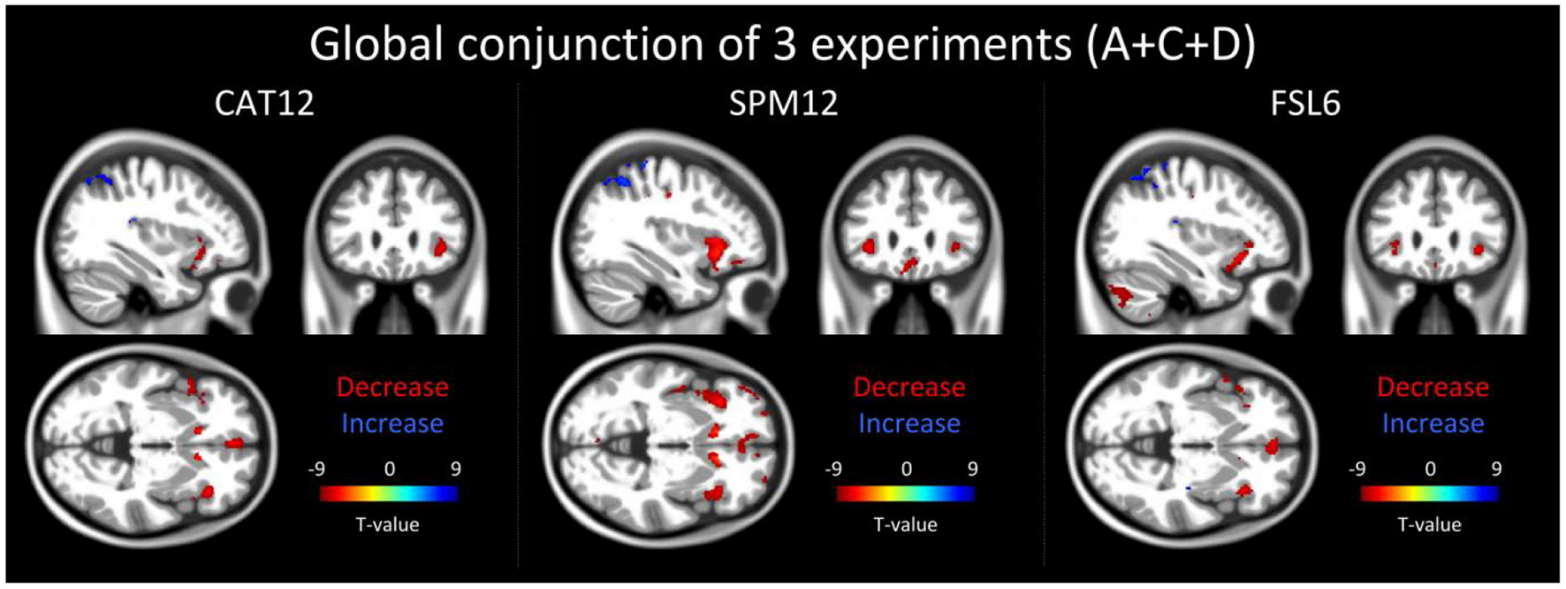
The result of the global conjunction test across all three reported experiments (20 participants for each experiment) is shown in 3 orthogonal slices (xyz−34 27−2 mm) for three different software packages: CAT12, SPM12 and FSL6. Global conjunction tests where the differences between the time points were consistently high and jointly significant across all three experiments (A+C+D) using a minimum statistic. Decreases in gray matter of structural magnetic resonance images are displayed in red colors while increases are indicated by blue colors.

Using CAT12 we found reduced local gray matter across all three experiments bilaterally in the caudate, the insula, the inferior frontal and the cingulate gyrus. Larger gray matter volume was found bilaterally in the parietal lobe and the precuneus, and in the left occipital gyrus (Figure 3).

Analysis of the data preprocessed with SPM12 and FSL6 showed a large overlap to the global conjunction result of CAT12 and similar regions could be detected (Figure 3).

**Functional MRI in Project A:** The group analysis for the repetitive painful electrical stimulation showed a significant activation (*p* < 0.05 FWE-corrected) in brain regions associated with pain processing. These areas comprise insular cortex bilaterally, midcingulate cortex, dorsolateral prefrontal cortex (DLPFC) secondary somatosensory cortex (SII), thalamus and cerebellum. Since we learned through Projects B-D that a nociceptive input plays no role to explain the structural changes we did not use these functional data in any of the above analysis.

## Discussion

Our main finding is an exceptionally robust, yet locally distinctive decrease in local gray matter T1-signal after short-term repetitive MPRAGE imaging in healthy volunteers. These changes were mostly located in the anterior and rostral cingulate cortex and both insula cortices. This finding could be reproduced in 3 different cohorts, including 96 scanning sessions and 72 healthy volunteers on two different 3 Tesla scanners. Additionally, we also identified increases in local gray matter volume between subsequent scans. Again, these effects were stable over the different cohorts, but included areas were more threshold dependent, compared to the found decreases in GMV.

Opposed to the schedule of longitudinal studies, which usually comprise several weeks or even months our study focuses on short-term repetitive MPRAGE acquisition. Our finding resulted from a repetitive MPRAGE image acquisition time frame of just approximately 30 min separated by either a simple 15 min pain task (project A) or “no specific task” (projects B-D) in healthy volunteers. We originally interpreted these changes to be caused by, or secondary to, the pain stimulation in the first cohort (project A). The reason for this was that these changes matched perfectly with BOLD fMRI data under pain stimulation (20, 21) and the anatomical location of these changes was within distinct central pain processing areas (22) (Figure 3). Much to our surprise we observed the same effect in the *post-hoc* scanned control cohorts (projects C and D) with identical scanning protocol but without any pain stimulation. This finding argues strongly against pain being causative for the observed changes. The observed results could only be detected within a specific scanning session if an EPI or REST (i.e. independent of a stimulus presentation) sequence was run before the second T_1_-weighted image. The effect was however more pronounced in EPI sequenced sessions than in the rest sessions, which suggests that future morphometric studies that are designed as T_1_-weighted images in combination with a functional MRI scanning session should focus on a consistent timing at which time-point during the experiment the T_1_-weighted scan (before or after the EPI sequences session) is conducted.

While temporal instabilities of the MR systems used as the source of the observations cannot be definitely excluded, this seems rather unlikely for several reasons: Firstly, two different MR systems with different gradient hardware and at different sites were involved. Second, most system instabilities, e.g. due to gradient and shim heating, are related to, or at least more pronounced for, severe hardware demands like EPI measurements, but in the present study differences were also observed without any measurements in-between. Third, the regional effects reported are quite localized while the scanner hardware (RF chain, gradient and shim hardware) presumably would cause more global changes.

Due to the short time frame a biological interpretation of our results is challenging. Possible explanations could be hemodynamic changes in blood flow (23) and increased blood pressure (17, 24) caused by the subjects' perception in the scanner. Data from neuropsychological studies investigating the effect of anxiety disorders on gray matter changes further support this assumption (25–27). Other reasonable explanations comprise possible fluid shift between extra- and intracellular spaces. When taking the relatively low specific absorption rate (SAR) into account, warming induced by radio-frequency electromagnetic fields utilized to acquire images (28) can be discarded as a possible explanation. We also conclude that the rating sequences applied could not result in the GMV changes we found as they are distributed specifically in brain networks associated with pain. Biological effects other than thermal caused by radiofrequency electromagnetic fields are complex and still highly speculative (29). Whether the respective regions such as anterior cingulum and insula are especially susceptible for magnetic or radiofrequency influence therefore explaining our results should be addressed in future studies. We found no literature on the interaction of specific absorption rate (SAR) on findings using voxel based morphometry or statistical parametric mapping (SPM).

However, the potential influence of the magnetic fields (static and time-varying) on brain function has been discussed since the late nineties (30). One might speculate that the rather local signal effects in our study may be a result of the geometrical distribution of the neuronal tracts in the various cortical brain regions being differently susceptible to the rapidly changing magnetic fields. There is some evidence that in another technique using rapidly changing magnetic fields, i.e. transcranial magnetic stimulation (TMS), the effects on neuronal activity are orientation dependent (31).

A decrease in gray matter estimations between the first and the subsequent conditions can be interpreted as a decline from baseline or alternatively as a return to baseline after prior signal enhancement. One could speculate that the changes that we see between scan S_HH1_ vs. scan S_HH2_ after noxious input are triggered by expectation, i.e. we actually describe an apparent “activation” (in the broader sense of a biological signal) or “increase” in brain areas before the experiment as volunteers anticipated the pain paradigm. However, when comparing the scan (project A, S_HH1_) before the experiment with the scan of the *post-hoc* experiment (project B, S_HH3_) (involving no expectation as the volunteers were told that no noxious input would follow), no significant change in the GMV at the defined thresholds could be observed. Our interpretation is that the respective changes of the T_1_-signal are reversible and widely rules out a putative bias introduced by expectation or conditioning that could have influenced our results.

We are fully aware that the results of our study have to be interpreted with great caution. What gives strength to our data is the fact that the results were very robust and could be reproduced in 3 independent projects (A/C/D) including almost 100 scanning sessions and survived very conservative statistical correction (FWE, *p* < 0.05) in all analyses. As the protocols were done on different scanner models using different head coils we can for the most part rule out a probable scanner-specific bias. Moreover, the preprocessing is using an inverse-consistent registration approach that guarantees independence from the order of selecting the time points. This was an issue of older approaches where the baseline image was used as reference for the registration and therefore no interpolation was necessary for this baseline image, which led to unbalanced interpolation effects between the time points. In order to address and test that our longitudinal preprocessing is not dependent on the order of time-points we changed the selection order for data from Experiment D and found no dependency from this order. This further demonstrates the inverse-consistency of our preprocessing. Additionally, we tested the global conjunction across all three experiments using a minimum statistic in SPM12, which resulted in similar regions that were found for the three experiments separately. Although the extent and the location of the findings differ between the experiments, we could find a pattern that is somewhat consistent across all experiments. Moreover, a similar pattern in the global conjunction analysis could also be observed when we additionally used two different VBM pipelines of SPM12 and FSL6. This emphasizes that the observed effects are not a simple artifact due to the processing pipeline used.

Whatever the biological/methodological interpretation underlying the GMV change, we note that these reductions were definitely not randomly distributed but only observed in specific areas and are exceptionally robust even over different cohorts and different scanners.

We note that this may have serious implications for published and future studies especially in (but not limited to) those that are designed as T_1_-weighted images in combination with a functional MRI scanning session. We propose that all future studies investigating T_1_-signal changes between cohorts but especially in longitudinal experimental designs (i.e. “brain plasticity studies”) should focus on and specifically report a consistent timing at which time-point during the experiment the T_1_-weighted scan is conducted. Additionally, this study also addresses the imperative necessity of a control group (with identical timing parameters) for longitudinal imaging studies to monitor for a possible systematic scanner – time effect influencing T_1_-signal.

## Conclusions

The current study suggests that repetitive short-term MPRAGE imaging can show significant T_1_-signal changes resulting in altered GMV estimations in highly specific and circumscribed brain areas. Although we can only speculate about the underlying mechanisms, the robustness of the results is striking. We would like to draw attention to the necessity of a careful order and timing of MR protocols and paradigms in longitudinal VBM studies.

## Data Availability Statement

Study data supporting the findings of this study are available from the corresponding author upon reasonable request after ethics approval and receipt of a signed material transfer agreement.

## Ethics Statement

The studies involving human participants were reviewed and approved by Ärztekammer Hamburg PV3562. The patients/participants provided their written informed consent to participate in this study.

## Author Contributions

GB: data collection, data analysis, data interpretation, and writing the manuscript. IE: data collection, data interpretation, and revising the manuscript. MM and CG: data analysis, data interpretation, and revising the manuscript. FF and MV: data collection, data analysis, and revising the manuscript. AM: conception and design of the study, data interpretation, and revising the manuscript. All authors contributed to the article and approved the submitted version.

## Funding

This work was supported by the German Research Foundation, SFB936/A5 (AM).

## Conflict of Interest

The authors declare that the research was conducted in the absence of any commercial or financial relationships that could be construed as a potential conflict of interest.

## Publisher's Note

All claims expressed in this article are solely those of the authors and do not necessarily represent those of their affiliated organizations, or those of the publisher, the editors and the reviewers. Any product that may be evaluated in this article, or claim that may be made by its manufacturer, is not guaranteed or endorsed by the publisher.
